# Trophic niche adaptation of mountain frogs around the Sichuan Basin: individual specialization and response to climate variations

**DOI:** 10.1186/s12983-024-00553-z

**Published:** 2024-12-19

**Authors:** Kaiyue Cui, Shengnan Yang, Junhua Hu

**Affiliations:** 1 Chengdu Institute of Biology, Chinese Academy of Sciences, No. 23, Qunxian South Road, Tianfu New Area, Chengdu, 610213 China; 2 University of Chinese Academy of Sciences, Beijing, 100049 China

**Keywords:** Ecological adaptation, Niche shift, Ontogenetic, *Quasipaa boulengeri*, Stable isotopes, Specialist

## Abstract

**Background:**

Climatic and geographic variations have profound effects on the resource utilization of individuals and populations. Evaluating resource use in different environments is crucial for understanding species ecological adaptation strategies and promoting biodiversity conservation. Stable isotopes are widely used to assess trophic niches, providing quantitative indicators of ecological interactions between organisms and resource use in ecosystems. This study assesses the trophic niche traits of spiny-bellied frogs (*Quasipaa boulengeri*) in the marginal mountains of the Sichuan Basin in southwestern China using stable isotopes. Trophic niche variation under different time periods and environmental conditions is explored.

**Results:**

The spiny-bellied frogs experienced a significant reduction in trophic niche width during the past breeding season. The populations in the northwestern Sichuan Basin had a greater trophic niche width than the southeastern populations, and their δ^15^N values showed a positive correlation with temperature seasonality and a negative correlation with annual precipitation. Despite differences between the northwestern and southeastern populations, there was a consistent trend of increased individual specialization with latitude in both the northwestern and southeastern regions.

**Conclusions:**

Ontogenetic niche shifts and differences in trophic niche traits between the northwestern and southeastern populations indicate diverse adaptation strategies in mountain frogs. The findings underscore the impact of geographical and climate variations on the resource utilization of amphibians. In addition, patterns of individual specialization highlight the significance of considering intra- and interpopulational changes when studying ecological adaptation.

**Supplementary Information:**

The online version contains supplementary material available at 10.1186/s12983-024-00553-z.

## Background

The trophic niche is a crucial component of ecological niche theory because it represents the trophic interactions between organisms within an ecosystem [1, 2]. These interactions are essential for assessing population dynamics, which involve changes in the size, composition, and distribution of populations over time [3–5]. The trophic niche is influenced by various factors, such as species characteristics, resource availability, and environmental changes [5–7]. Characterizing the trophic niches of species within and among populations can provide vital insights into understanding the ecological adaptations of species. Wildlife often exhibits substantial intraspecific variation in resource utilization across temporal and spatial scales, and demonstrates extensive individual trophic specialization within populations [7–9]. These variations play a crucial role in shaping population niche widths and have significant implications for species adaptation and evolution [3, 5]. According to the niche variation hypothesis, population niche expansion is primarily driven by increasing variation among individuals [10]. Therefore, compared to populations with narrower niches, populations with broader dietary niches should exhibit greater individual variation in resource utilization, demonstrating individual specialization within the population.

The niche variation hypothesis emphasizes the importance of considering dietary variance as a direct and pertinent measure for assessing resource use patterns [11, 12]. Previous studies have often relied on direct observations, such as analyzing gut/stomach contents, or measuring phenotypic variations in traits related to food utilization, to quantify individual dietary changes [1, 6, 13]. However, these methods either capture only transient food use patterns or are closely tied to behavioral or physiological characteristics [13, 14]. Stable isotope analysis (e.g., carbon and nitrogen stable isotopes) has been widely used to describe consumer-resource relationships and can assess individual-level trophic changes over periods ranging from weeks to years [1, 5, 15]. Carbon stable isotopes (δ^13^C) remain relatively constant across trophic levels and provide information on consumer food sources; nitrogen stable isotopes (δ^15^N) exhibit progressive enrichment in the food chain and typically reflect the trophic position of predators [1, 16]. For example, through an analysis of stable isotope signatures in coexisting anurans, researchers found that larger individuals generally feeding at higher trophic levels, and species with enriched carbon isotope ratios usually foraged farther from ponds [15]. Therefore, δ^13^C and δ^15^N are ideal indicators for characterizing trophic niches within and among populations. Such a focus on trophic variation offers a robust framework for exploring the complexities of evolving resource exploitation, as envisioned by the niche variation hypothesis.

**Fig. 1:**
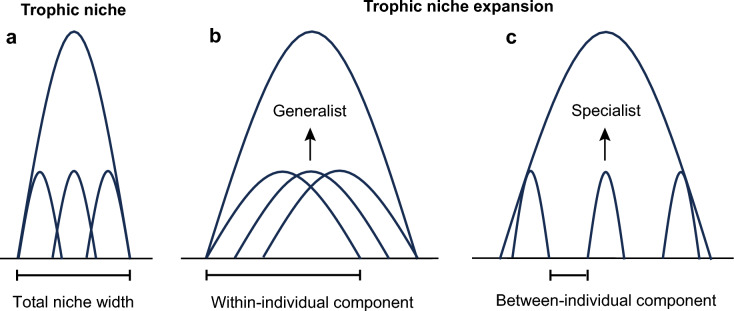
Trophic niche components and the expansion pattern. **a** The total niche width of a population is attributed to the within-individual component and the between-individual component. **b-c** Both the intraindividual and interindividual variations in resource use can contribute to trophic niche expansion

Stable isotope ratios have been widely used to describe individual trophic specialization across various taxa, as they can quantify variation at both the individual and population levels along resource axes [17, 18]. Individual specialization refers to the extent or diversity of resource utilization by individuals within a population [11, 14]. The total niche width of a population can be divided into two components: the within-individual component and the between-individual component (Fig. 1) [14, 19]. The within-individual component represents the average variance in resource utilization within individuals, while the between-individual component represents the variation between individuals [11, 14, 19]. The degree of individual specialization is commonly measured by the ratio of within-individual component/total niche width [14]. A ratio approaching 1 suggests that individuals utilize most of the available resource and exhibit low individual specialization. Conversely, a ratio approaching 0 indicates a high degree of individual specialization, with individuals exploiting a narrower range of resources specific to their unique ecological roles. Ecological interactions may influence variations in both within-individual components and between-individual components [5]. Researchers have analyzed how biotic and abiotic factors affect population niches and the degree of individual specialization in assemblages [5, 11, 12, 20]. However, patterns of individual specialization within species remain largely unknown [5, 11].

Amphibians are considered the most endangered group of vertebrates, suffering from species extinction and population decline worldwide [21]. Characterizing trophic niches is crucial for comprehending their ecological adaptive strategies and developing effective conservation measures. Ontogenetic niche shifts refer to variations in ecological roles and resource use observed within a population throughout different life stages [8]. The dietary requirements and ecological roles of amphibians change in response to environmental and individual conditions [22]. In addition, their morphological characteristics limit their resource acquisition [23]. For instance, smaller frogs can consume only smaller prey, resulting in a narrower niche width. In contrast, larger frogs feed on both smaller and larger prey, thereby increasing their ecological niche width [15, 24]. Considering the low mobility and highly permeable skin of amphibians, temperature and water availability have profound influences on the ecological adaptation of these frogs [23, 25, 26]. In response to these changes, amphibians may modify their feeding habits and foraging areas [27]. Although amphibians have demonstrated the ability to adapt to environmental changes, it is unclear how trophic niches change at different life stages and under varying environmental conditions.

In this study, we investigated wild spiny-bellied frog (*Quasipaa boulengeri*) populations and explored the effects of individual development and environmental changes on their trophic niche. The spiny-bellied frog is a mountain frog species found in southern China [28]. Wild populations of the spiny-bellied frog are declining due to overharvesting and habitat degradation [28]. Furthermore, climate change presents new threats to wild populations [29]. By quantifying the stable isotope traits of mountain frogs, we addressed the following questions: (1) Does the trophic niche change during frog growth? (2) How does the trophic niche respond to climatic factors? (3) What is the trend of individual specialization in these mountain frogs? We anticipate that the trophic niches of spiny-bellied frogs exhibit temporal variation during different life stages, with trophic niche traits responding to climatic factors. Furthermore, individual specialization is likely to vary across different geographic populations. We aimed to clarify the patterns of trophic niche variation and individual specialization, identify the relationships between trophic niches and environmental factors, and enhance our understanding of the ecological adaptations of amphibians.

## Methods

### Study area

**Fig. 2:**
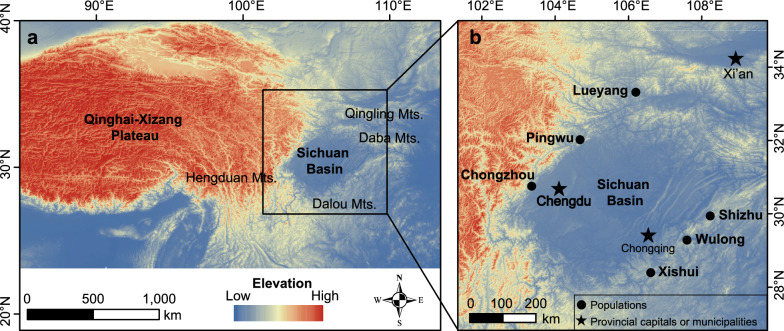
Geographic location of the spiny-bellied frog populations. **a** Location of the Sichuan Basin and the surrounding terrain. **b** Distributions of the northwestern and southeastern populations of the spiny-bellied frog

The study area is located in the marginal mountains of the Sichuan Basin in Southwest China (Fig. 2a). Due to the orogenesis of the Qinghai‒Xizang Plateau, western China has a highly complex mountain system [30]. The uplift of the eastern Qinghai‒Xizang Plateau impacted the mountains in the northern and western Sichuan Basin [31]. Considering the geographic variation and climate-driven shifts, we selected six populations of spiny-bellied frogs in the northwestern and southeastern Sichuan Basin (Fig. 2b).

### Sample collection

During June and July 2019, we conducted searches for frogs after sunset in and around streams using flashlights. Variations in metabolic rates among different tissue types result in differing isotopic turnover rates, enabling the isotopic traits of various tissues to reflect trophic niches across different temporal windows [32, 33]. In amphibians, muscle tissue reflects resource utilization over the weeks to months prior to sample collection, while bone collagen integrates the assimilation of food resources over years [32, 34]. We obtained a total of 42 adult frogs from six populations (Additional file 1: Table S1). For each frog, we extracted fourth toe and muscle tissues after euthanasia. All the samples were stored in 2 ml tubes, refrigerated in the field, and then stored at –20 °C in the laboratory. To obtain collagen, we used tweezers to remove the skin of the toes, and separated the ligaments and tendons from the bone. The bone samples were demineralized in 0.5 mol/L hydrochloric acid in a refrigerator for 24 h, after which they were dried in an oven at 60 °C for 48 h. Lipid extraction was then performed on bone samples using a 1:2 chloroform:methanol solution for 24 h, and this process was repeated several times. Finally, the samples were washed with deionized water and heated at 90 °C for approximately 12 h [35].

### Stable isotope analysis

The muscle and bone collagen samples were freeze-dried for 36 h until they reached a constant weight and then crushed into a fine powder. Subsamples weighing between 1–4 mg were analysed by loading them into tin capsules for carbon and nitrogen isotopic measurements. We used a vario isotope cube elemental analyser (vario ISOTOPE cube, Elementar, Germany) and an isotope ratio mass spectrometer (IsoPrimer 100, Isoprime, UK) to conduct the analysis. The isotope ratios were expressed as δ^13^C and δ^15^N using the following equation: δX = [(R_sample_/R_standard_) –1] × 1000, where X is ^13^C or ^15^N. R_sample_ and R_standard_ are the ^13^C/^12^C and ^15^N/^14^N ratios of the sample and standard, respectively. The laboratory standard was calibrated based on a set of international standards [36]. For nitrogen, the standard was atmospheric N_2_, and for carbon, the standard was a marine limestone known as Peedee Belemnite.

### Statistical analysis

After conducting Shapiro‒Wilk tests for normality and homoscedasticity, we used Student’s t-test to analyze the difference of δ^13^C and δ^15^N values between muscle and bone collagen. For population-level comparisons, we used analysis of variance (ANOVA). Using bivariate carbon and nitrogen data, we determined the spatial location of each individual to represent its trophic niche by employing means and covariance matrices [16]. To analyze ontogenetic niche shifts, we used the muscle and bone collagen to represent different time periods [32–34]. We then used the SIBER package to quantify trophic niche indicators of each tissue and population, where niche width is defined as the total area of the convex hull encompassing the data points, the standard ellipse area, and the sample size-corrected standard ellipse area [16].

We downloaded climate data from the CHELSA database [37] at a horizontal resolution of 30 arcsec (~ 1 km). We estimated the population range by creating a 10 km-radius buffer around each sampling point, taking into account the population ranges and dispersal abilities of amphibians [38]. We then randomly extracted climate factor data using “sample” in ArcGIS 10.1 (ESRI, Redlands, CA, USA). We conducted autocorrelation tests on 19 climate factors. After excluding factors with a correlation greater than 0.7 [39], we retained four climate factors related to the reproduction and growth of amphibians: mean annual air temperature, temperature seasonality, annual precipitation amount, and precipitation seasonality. Since the climate data did not follow a normal distribution (Shapiro test), we used Spearman correlation analysis to examine the relationships between the selected factors and the δ^13^C and δ^15^N values.

To quantify dietary specialization in spiny-bellied frog populations, we used the δ^15^N values of two tissues (muscle and bone collagen) as continuous data to calculate individual specialization using the RInSp package [40]. Niche expansion is observed when a greater total niche width correlates with increased individual specialization, as indicated by a low ratio of within-individual component/total niche widths [14, 19].

All the statistical procedures were implemented in R 4.2.1. The significance level was set at *p* = 0.05. Graphical output was generated using the ggplot2 package in R and Origin 2018 [41, 42].

## Results

### Trophic niche traits

**Fig. 3:**
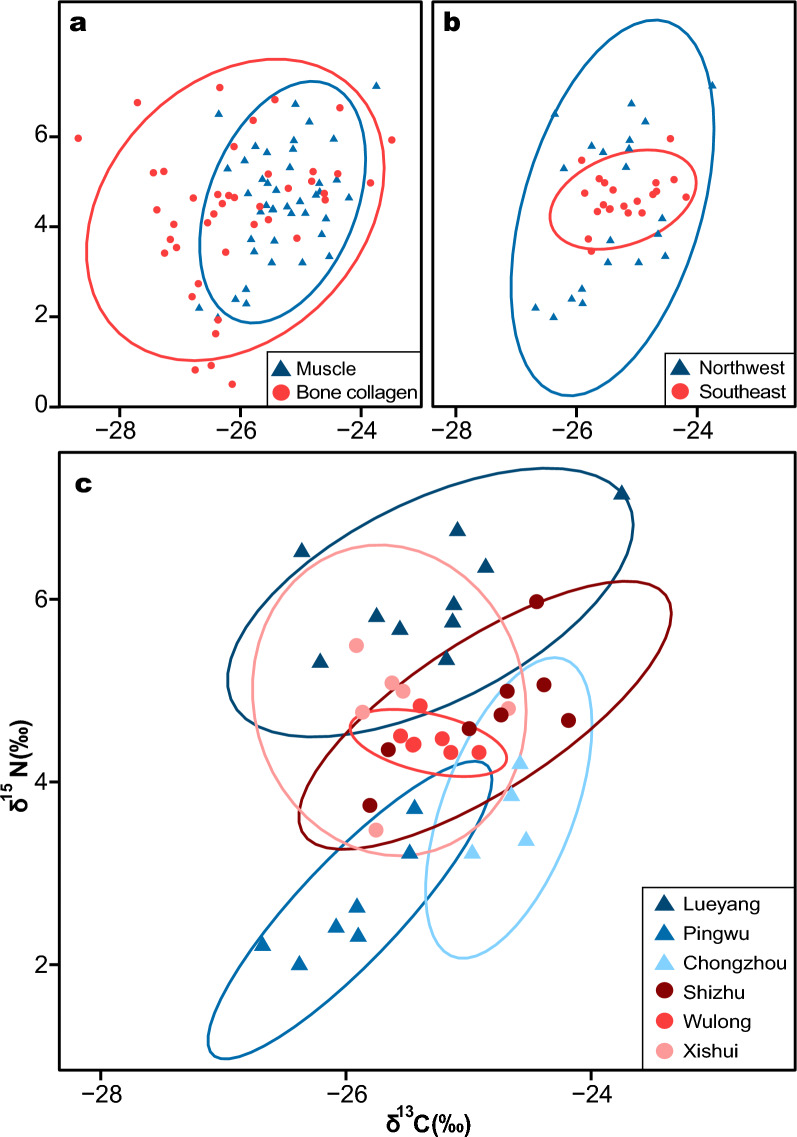
Isotopic biplots illustrating the ontogenetic niche shift and trophic variation in populations. The corrected standard ellipse areas (95% credible interval) of the different groups were plotted

The δ^13^C values were significantly greater in muscle tissues (mean ± SE, − 25.32 ± 0.10) than in bone collagen (− 26.09 ± 0.17; Table 1). Notably, muscle exhibited a narrower δ^13^C range than did bone collagen. The δ^13^C and δ^15^N values of muscle varied significantly among the six populations (Table 1). Trophic niche comparisons between tissues revealed a smaller niche width for muscle than for bone collagen (Table 2; Fig. 3a). After controlling for the sample size, the trophic niche width was greater in the northwestern populations than in the southeastern populations (Fig. 3b). The trophic niche width of the southeastern populations showed aggregation, whereas that of the northwestern populations tended to decrease (Fig. 3c).

**Table 1:** Comparisons of stable isotopic traits (δ^13^C and δ^15^N) between tissues and among populations

	Student’s t test			ANOVA test	
	t	df	*p*	F	*p*
Tissue					
δ^13^C (‰)	**3.92**	**65.64**	** < 0.001**	–	–
δ^15^N (‰)	0.60	77.63	0.55	–	–
Population					
δ^13^C (‰)	–	–	–	**4.77**	**0.002**
δ^15^N (‰)	–	–	–	**32.33**	** < 0.001**

Significant results are highlighted in bold. *p* = 0.05

**Table 2:** Trophic niche indicators for tissues and populations. TA: total area of the isotopic niche; SEA: standard ellipse area; SEA_C_: corrected standard ellipse area; WIC/TNW: within-individual component/total niche width of a population, indicating the degree of individual specialization

	TA (‰^2^)	SEA (‰^2^)	SEA_C_ (‰^2^)	WIC/TNW
Tissue				
Muscle	9.70	2.30	2.36	–
Bone collagen	19.61	5.38	5.52	–
Population				
Lueyang	2.54	1.20	1.34	0.33
Pingwu	0.19	0.24	0.36	0.36
Chongzhou	0.70	0.44	0.52	0.57
Wulong	0.15	0.11	0.13	0.48
Xishui	1.20	0.98	1.23	0.80
Shizhu	1.42	0.81	0.94	0.12

### Response to climate variations

**Fig. 4:**
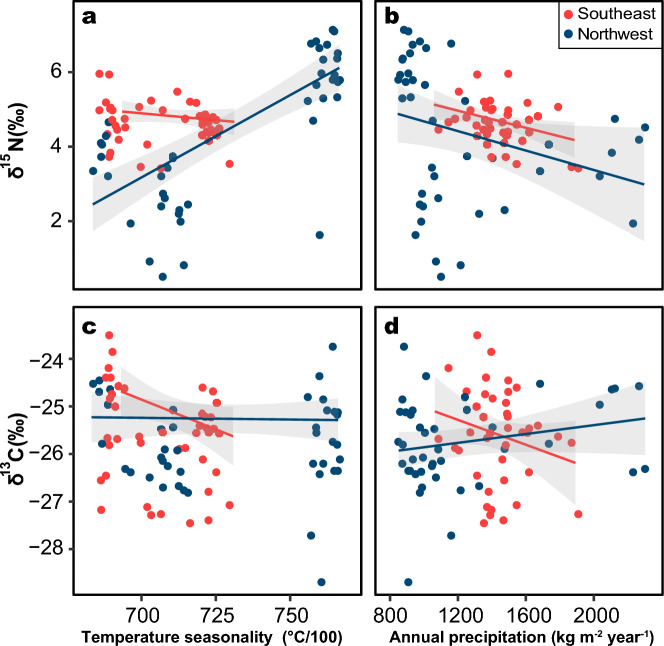
Relationships between stable isotope values (δ^13^C and δ^15^N) and climate factors (temperature seasonality and annual precipitation)

The δ^15^N value was significantly correlated with climatic factors in the northwestern populations. Specifically, there was a strong positive correlation with temperature seasonality and a strong negative correlation with the annual precipitation amount (Fig. 4a, b, Additional file 1: Table S2). The δ^13^C and δ^15^N values of the northwestern populations were significantly negatively correlated with precipitation seasonality. The δ^13^C values of the northwestern populations displayed nonsignificant correlations with climate factors, such as the mean annual air temperature, temperature seasonality, and annual precipitation amount. For the southeastern populations, the correlations between climate factors and stable isotope values (both δ^13^C and δ^15^N) were generally not significant (Fig. 4, Additional file 1: Table S2).

### Patterns of individual specialization

**Fig. 5:**
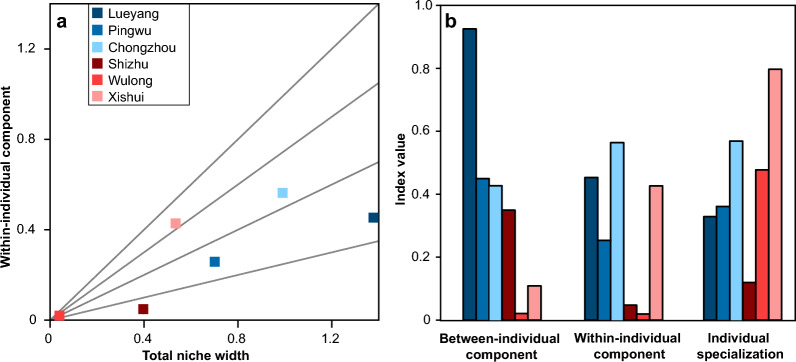
Trophic niche indices of the individual specialization of the northwestern and southeastern populations. **a** Relationship between within-individual component and population niche width. The four function lines indicate the ratio of within-individual component/total niche width. **b** Individual specialization comparison among six populations using δ^15^N data from muscles and bone collagens. The between-individual component, within-individual component and individual specialization of the six populations are shown. Shades of blue represent the three populations in the northwest, and shades of red represent the three populations in the southeast

For the northwestern populations, Lueyang and Pingwu had larger between-individual component values than within-individual component values, and the Chongzhou population had larger within-individual component values (Fig. 5). For the southeastern populations, the Wulong population had equal values for both the within-individual component and between-individual component (Fig. 5). Shizhu exhibited larger between-individual component values compared to within-individual component values, and the Xishui population had larger within-individual component values (Fig. 5). In both the northwestern and southeastern Sichuan Basins, the degree of individual specialization increased with latitude (Table 2; Fig. 5). In the northwest, Lueyang and Pingwu were both specialist populations. The southeastern populations showed more variable patterns of individual specialization, in which Shizhu was a specialist population and Xishui was a generalist population.

## Discussion

The trophic niche is an important indicator for assessing resource utilization and population dynamics [2, 7]. We used stable isotope analysis to investigate how mountain frogs use resources at different life stages and under different environmental conditions. Our results indicate that individuals undergo niche transfer during growth, resulting in a narrower trophic niche. The trophic niche traits, δ^13^C and δ^15^N, in relation to climatic factors and the degree of individual specialization show different patterns across geographic populations, suggesting that there are diverse ecological adaptation strategies within the species.

### Ontogenetic niche shift

The variations in the trophic niche width between muscle and bone collagen were pronounced mainly for δ^13^C, while there was no significant difference in δ^15^N (Fig. 3a). Different tissue types (e.g., muscle, bone collagen) have different turnover rates, providing the integration of resource utilization across different time scales [32, 34]. Muscle tissue typically reflects the consumer’s resource utilization over weeks to months, while bone collagen integrates information about food assimilation over several years [32, 33]. If predators have different diets, their isotopic characteristics will also exhibit variation. The δ^13^C signatures of predators are typically similar to those of the food they consume [1, 15]. The substantial variations in the δ^13^C values suggest that the diet structure of these frogs may have changed [43]. Consequently, our findings imply that spiny-bellied frogs may have recently acquired and assimilated resources from habitats with similar primary producers, resulting in a smaller trophic niche width [1, 16]. One of the carbon sources in streams is leaf litter and animal carcasses [44]. The δ^13^C values in the aquatic environment are lower than those in the terrestrial environment [15, 44]. Compared to bone collagen, the muscle tissue of spiny-bellied frogs has higher δ^13^C values, possibly due to the reliance on more terrestrial food resources during the breeding season. Spiny-bellied frogs breed from May to August [28], during which time they are frequently active along the shore and likely consume more land-based insects.

### Geographic variation and response to climate factors

Our study revealed significant differences in the trophic niche widths between the northwestern and southeastern populations of spiny-bellied frogs around the Sichuan Basin (Fig. 3b). The northwestern populations exhibit broader trophic niche widths compared to their southeastern counterparts, reflecting greater diversity and availability of food resources [43, 45]. The three southeastern populations showed high overlap in trophic niches (Fig. 3c), indicating more concentrated diet structures (δ^13^C) and trophic levels (δ^15^N). The varied topography in these regions may impact ecological conditions and resource availability. In addition, climate factors strongly influenced δ^15^N in mountain frogs (Fig. 4; Additional file 1, Table S2). In the northwestern populations, δ^15^N values were positively correlated with temperature and precipitation (Fig. 4). Regional biogeochemical processes, such as nutrient availability, temperature, and precipitation, can contribute to additional isotopic variation in consumers by influencing isotopic baselines and physiological condition of organisms [46]. For instance, climate change-induced alterations in nitrogen deposition in primary producers could affect δ^15^N enrichment in food chain [47]. Furthermore, the southeastern part of the basin receives abundant precipitation, which, combined with slight seasonal temperature variations, provides a more stable environment. This stability may result in a more consistent supply of resources, leading to more concentrated trophic niches.

### Individual specialization patterns

Individual specialization plays a crucial role in shaping food web dynamics [14, 48]. Individual specialization increased with latitude in the northwestern and southeastern populations (Fig. 5). Latitude is a composite variable intricately linked to numerous biotic and abiotic factors [49]. Environmental heterogeneity leads to niche variations both within and between populations, as interactions involving prey availability, competitive pressures, and physiological stresses influence foraging strategies [8]. Diminished resource availability can lead to niche expansion and heightened individual specialization of populations [15, 50]. Conversely, resource diversity may create ecological opportunities for expanding trophic niches and individual specialization [15]. Therefore, the results may suggest a more diverse pattern of resource use in higher-latitude populations [18, 43]. However, the two geographic groups exhibited differences in individual specialization patterns (Table 2; Fig. 5). Two of the three northwestern populations were specialist populations, while the southeastern populations included one generalist population and one specialist population. Niche expansion at the population level (i.e., increasing total niche width) can be reflected at the individual level through three main pathways: increasing within-individual components, increasing between-individual components, or increasing both within-individual components and between-individual components [5]. The generalist population in the southeastern region (Xishui) had more within-individual components than between-individual components, while the specialist population (Shizhu) had more between-individual components, suggesting diverse ecological adaptation strategies in the southeastern Sichuan Basin. The niche variation hypothesis postulates that populations with broader niches should exhibit greater interindividual dietary variation or individual specialization than more constrained populations [11, 12]. Our findings show that an increase in the between-individual component can accompany an expansion in the trophic niche (e.g., Lueyang, Fig. 5). The niche expansion can also be dominated by an increase in within-individual components (e.g., Xishui, Fig. 5) [5]. The consequences of individual specialization are usually discussed in the context of intraspecific competition [14]. Intraspecific niche variation manifests in various forms, including differences between individual life stages, sexes, and among individuals [8, 11, 45]. Resource use differentiation within populations across varying environments can increase population stability through combinatorial forms or by reducing intraspecific competition [15, 50, 51]. Contrary to the trend towards generalist populations, our results underscore the unique ecological roles of mountain frogs, emphasizing the need for more focused research in trophic ecology to understand their ecological adaptation strategies.

## Conclusions

Our study used stable isotope technology to understand the dynamic and adaptive strategies of trophic niche traits in mountain frogs. The trophic niche width of the frogs shifted significantly at different life stages. The δ^15^N values showed significant variations that correlated with changes in temperature and precipitation. In both the northwestern and southeastern populations, an increase in latitude corresponded to a greater degree of individual specialization. This study explored the influence of geographic and climatic variations on trophic niches, highlighting the importance of considering individual variation when studying ecological adaptations. As climate change continues to modify the ecological environment, quantifying trophic niche traits can provide theoretical experience and scientific support for assessing the adaptive capacity of wildlife and ecosystem stability.

## Supplementary Information

Additional file1

## Data Availability

The datasets generated and/or analyzed during the current study are available in the figshare repository, 10.6084/m9.figshare.26064733.
