# Supplementary material for: Trophic niche adaptation of mountain frogs around the Sichuan Basin: individual specialization and response to climate variations
Source: Front Zool. 2024 Dec 19;21:32. doi: 10.1186/s12983-024-00553-z (PMC11657682; doi:10.1186/s12983-024-00553-z)
Supplement: Supplementary file 1 — Additional file1 [file 12983_2024_553_MOESM1_ESM.docx]

Table S1. Detailed information for the collected populations. Populations are named after the county-level cities. N means the number of collected individuals.

| Population | Province | Longitude and latitude | N | Region |
| --- | --- | --- | --- | --- |
| Lueyang | Shaanxi | 33.34° N 106.19° E | 10 | Northwest |
| Pingwu | Sichuan | 32.03° N 104.68° E | 4 | Northwest |
| Chongzhou | Sichuan | 30.77° N 103.36° E | 7 | Northwest |
| Shizhu | Chongqing | 29.94° N 108.22° E | 8 | Southeast |
| Wulong | Chongqing | 29.28° N 107.59° E | 7 | Southeast |
| Xishui | Guizhou | 28.40° N 106.59° E | 6 | Southeast |

Table S2. Relationships between stable isotope values (δ^13^C and δ^15^N) and climate factors (Bio 1: mean annual air temperature; Bio 4: temperature seasonality; Bio 12: annual precipitation amount, Bio15: precipitation seasonality).

|  | δ^13^C | |  | δ^15^N | |
| --- | --- | --- | --- | --- | --- |
|  | R | *p* |  | R | *p* |
| Northwest populations | | | | | |
| Bio 1 | -0.19 | 0.22 |  | 0.06 | 0.71 |
| Bio 4 | -0.06 | 0.68 |  | **0.63** | **<0.001** |
| Bio 12 | 0.10 | 0.55 |  | **-0.51** | **<0.001** |
| Bio 15 | **-0.37** | **0.02** |  | **-0.37** | **0.002** |
| Southeast populations | | | | | |
| Bio 1 | 0.17 | 0.28 |  | 0.17 | 0.29 |
| Bio 4 | -0.20 | 0.21 |  | -0.18 | 0.25 |
| Bio 12 | -0.11 | 0.51 |  | -0.25 | 0.11 |
| Bio 15 | -0.23 | 0.14 |  | -0.16 | 0.30 |

Significant results are highlighted in bolds. *p* = 0.05.
